# A review of 10 patients treated with the masquelet technique and microsurgical technique combined for Gustilo type III open tibial fractures

**DOI:** 10.1186/s12891-024-07478-y

**Published:** 2024-05-07

**Authors:** Jingxuan He, Xiaofeng Xia, Bing Zuo, Jiaguo Tang, Peng Wang

**Affiliations:** Department of Orthopedics, General Hospital of the Yangtze River Shipping, Wuhan city, Hubei province 430010 China

**Keywords:** Masquelet technique, Open comminuted fractures, Tibial bone defects, Microsurgical technique, Bone healing

## Abstract

**Background:**

Open tibial fractures often include severe bone loss and soft tissue defects and requires complex reconstructive operations. However, the optimal treatment is unclear.

**Methods:**

This retrospective study enrolled patients with Gustilo type III open tibial fractures from January 2018 to January 2021 to assess the clinical utility of Masquelet technique together with microsurgical technique as a combined strategy for the treatment of open tibial fractures. The demographics and clinical outcomes including bone union time, infection, nonunion and other complications were recorded for analysis. The bone recovery quality was evaluated by the AOFAS Ankle-Hindfoot Scale score and the Paley criteria.

**Results:**

We enrolled 10 patients, the mean age of the patients and length of bone defects were 31.7 years (range, 23–45 years) and 7.5 cm (range, 4.5–10 cm) respectively. Bone union was achieved for all patients, with an average healing time of 12.2 months (range, 11–16 months). Seven patients exhibited a bone healing time of less than 12 months, whereas 3 patients exhibited a bone healing time exceeding 12 months. No significant correlation was found between the length of bone loss and healing time. In addition, no deep infection or nonunion was observed, although 2 patients experienced wound fat liquefaction with exudates and 1 patient presented with a bloated skin flap. The average AOFAS Ankle-Hindfoot Scale score was 80.5 (range, 74–85), and all patients were evaluated as good or exellent based on the Paley criteria.

**Conclusions:**

Our study indicated that the use of the Masquelet technique and the microsurgical technique as a combined strategy is safe and effective for the treatment of Gustilo type III open tibial fractures.

## Background

Gustilo type III open fractures, frequently seen in traumatic injuries, often include severe bone defects or loss. Such fractures are accompanied by blood vessel rupture, skin and soft tissue defects, and bone exposure, which increase the complexity of the situation and the difficulty of surgical treatment [1]. The ilizarov bone transport technique, a method of distraction osteogenesis, has been widely adopted for two decades to treat bone losses larger than 40 mm [2, 3]. The alternate technique is to place a vascularized bone graft with blood vessels. Unfortunately, this technique often includes a long healing time and multitude of complications [4, 5]. Masquelet technique, which involves a typical two-stage procedure, after external fixation, a bone cement spacer is temporarily placed in the form of stringed beads to induce autologous membrane formation, which facilitates bone regeneration in stage I for a few weeks. The procedure is followed by bone-free nonvascularized autologous bone grafting harvested from iliac crests in stage II. Upon removal, the spacer leaves behind a bed of well-vascularized granulation lined bed, which has been noted to facilitate bone graft integration. By applying this two-stage technique, a number of studies have shown acceptable outcomes. However, failure cases with various causes have also been reported. Meng et al. reviewed 41 Masquelet technique studies with 677 patients and a total of 680 fractures and reported that 26.03% of the patients had complications-8.97% had nonunion, and 8.09% had deep infections, and the highest percentage of complications occured in the tibia [6]. Despite recent improvements in surgical techniques for treating Gustilo III open fractures, the incidence of failure has remained high, especially for patients with open fractures in the lower tibia due to poor blood supply and less muscle coverage in these areas [7].

Godina’s study revealed that the implementation of early microsurgical wound reconstruction led to a notable reduction in infection rates, as well as a shortened hospitalization period and accelerated bone healing [8]. To date, there are limited studies on the practical implementation of microsurgical techniques for the remediation of wounds with large bone loss, and the optimal strategy for addressing such severe injuries remains unclear [9].

In this study, we aimed to determine the safety and clinical effectiveness of the combination of the Masquelet technique and microsurgery technique for the treatment and reconstruction of Gustilo type III open tibial fractures caused by acute injuries through a retrospective review of 10 cases in a single center, and to share some tricks and tips to improve clinical outcomes.

## Methods

**Patients.** We retrospectively evaluated 10 patients (6 males and 4 females) who underwent surgery via the Masquelet technique plus microsurgery as a combined treatment between January 2018 and January 2021 at the General Hospital of the Yangtze River Shipping. The inclusion criteria were a tibia bone defect with an opened comminuted fracture, a bone defect length > 2.5 cm, an age of 18 to 50 years, time since an injury < 6 h, and a follow-up time > 12 months. Patients with diseases such as serious cardiovascular disease, diabetes, cerebrovascular disease, or other basic diseases were excluded. All patient data were retrieved from the hospital’s electronic case files and this study was approved by the hospital ethics committee. Informed consent to participate was obtained from patients.

### Surgical protocol

**Stage one.** After complete wound debridement, bone fragments with severe contamination were removed, while the larger bone fragments were retained. In this stage, composite external fixation frame was used for temporary fixation. **Stage two.** Bone cement, made up of polymethyl methacrylate (PMMA) and loaded with vancomycin, was prepared to fill the bone loss. Then, wound was closed to attain skin coverage if possible, and vacuum sealing drainage (VSD) for wound closure. As per local soft tissue restraints, local flaps were designed based on local vascular patterns or composite flaps based on muscles. Depending upon the lack of local pedical flaps, anterolateral femoral free flaps were used. The wounds were reevaluated at each dressing change, and a second-stage skin graft was made if necessary. **Stage three.** After the formation of the induced membrane (6–8 weeks), the bone cement was removed. Then, autologous iliac bone and allograft bone were implanted into the induction membrane to complete. the repair of bone defects. Remove the external fixator and replaced it with intramedullary nail fixation or intramedullary nail combined with locking plate fixation. **Tips and tricks**:1) Overall, the placement of cement spacers and the generation of an inducing membrane are crucial for stimulating bone regeneration and achieving desirable outcomes. 2) Ensure thorough debridement is conducted during the initial surgery with no signs of infection. 3) Strictly debride the defect and completely remove contaminated free bone fragments or necrotic tissue. 4) The edges of bone defects should be healthy with active bleeding. 5) Sufficient blood supply should be ensured to the surrounding soft tissues. 6) Adequate soft tissue coverage is indispensable. 7) Wound closure should be tension-free. 8) Cement spacers must be placed for a minimum of 6–8 weeks. 9) Prior to transplantation, bone biopsy and normal levels of ESR, white blood cell count, and CRP levels should be confirmed. 10) Ensure that the cement encapsulates both ends of the bone defect and extends approximately 1 cm beyond to ensure successful membrane formation later on. 11) Rinse bone cement with cold physiological saline during the surgical procedure to prevent heat damage to normal tissues surrounding the defect. 12) Adequate amounts of graft material should be provided according to the size of the defect. For large defects, autologous bone grafts can be combined with allografts or bone substitutes. 13) Sufficient mechanical stability must be provided, usually achieved with plate fixation, intramedullary nail fixation, or a combination of plates and intramedullary nails. The plates can be placed supra-membraneously to minimize disruption to the blood supply of the periosteum and ensure firm fixation beneath the membrane.

**Evaluation of recovery.** After wound healing, a follow-up evaluation was performed, which included a monthly review of the fracture untill it healed. The Paley classification was used to evaluate the quality of fracture healing [10]. The ankle posterior foot score of the American Orthopedic Foot and Ankle Association (AOFAS) was used to evaluate bone-function recovery [11]. Nonunion was conceptualized as the lack of bone union or the restricted advancement of union as observed through radiographic assessment after a minimum follow-up period of one year. Delayed union was defined as a duration of union exceeding one year [12].

### Statistical analysis

The relationships between the length of bone loss and healing time were evaluated using Spearman’s correlation analysis (*p* < 0.05 indicates statistical significance).

## Results

**Fig. 1:**
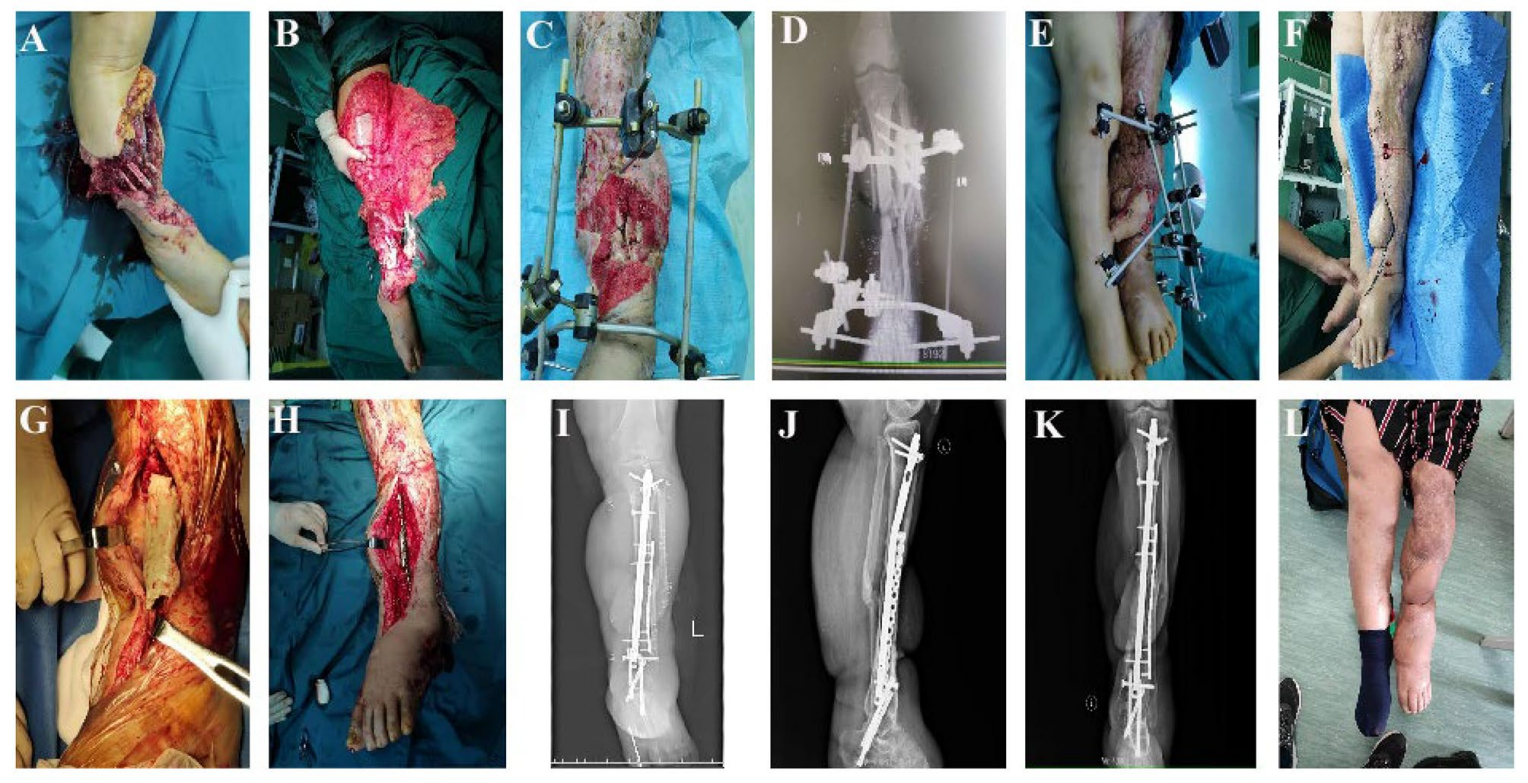
A 27-year-old female patient (case 6) with Gustilo IIIB open tibial fracture. **(A, B)** Photograph of wound and bone defects before operation. **(C, D)** After debridement, external fixation was performed. **(E)** the defect was filled with bone cement spacer, external fixation and soft tissue repair with Crossover posterior tibial artery perforator flap were performed. **(F-I)** 12 weeks later, cancellous bone grafting was performed **(J-L)** Radiograph and photograph of 12 months post implantation operation

Ten patients with open tibial fractures who underwent surgery via the Masquelet technique plus microsurgery as a combined treatment were evaluated in the present study. No deep infection was observed, and bone union was achieved for any of the patients. The average age of the patients was 31.7 years (range, 23–45 years), and the mean length of the bone defects was 7.5 cm (range, 4.5–10 cm). Among the 10 cases, 8 were caused by traffic accidents and the remaining 2 were caused by crush injuries. Moreover, 7 patients were classified as having Gustilo type IIIA fractures, while 3 were classified as type IIIB fractures. The minimum follow-up time was 20 months (mean 25 months, range 20–34 months) (Table 1). In the study, the mean wound area measured 115 cm^2^, with a range from 50 to 180 cm^2^. In the initial operation, all patients underwent temporary fixation using a composite external fixation frame. Soft tissue reconstruction procedures varied among patients: three received posterior tibial artery perforator flap local transposition transplantation, four underwent Sural nerve nutrient flap local transposition transplantation, two received Crossover posterior tibial artery perforator flap transplantation, and one patient underwent Free anterolateral thigh flap. In the subsequent operation for internal fixation, nine cases utilized Intramedullary nail plus locking steel plate combined fixation, while one case underwent Intramedullary nail internal fixation (Table 2). Through follow-up examination and evaluation, we found bone healing was achieved in all 10 patients, with an average healing time of 12.2 months (range, 11–16 months). Seven patients exhibited a bone healing time of less than 12 months, whereas three patients with a healing time exceeding 12 months were classified as having delayed union. The average AOFAS Ankle-Hindfoot Scale score was 80.5 (range, 74–85), and the bone healing of all patient was evaluated as excellent or good based on the Paley criteria. No significant correlation were found between bone loss length and healing time (*r*=-0.164, *P* = 0.651). In addition, two patients experienced wound fat liquefaction with exudates, but the wounds recovered well after timely dressing changes. A single patient exhibited edema in the skin flap, necessitating flap revision surgery following successful bone consolidation (Table 3). Figure 1 showcased both preoperative and postoperative photographs and radiographs depicting the condition of the wound and tibia.

**Table 1:** Patient demographics

Case	Gender/age	Injury factor	Bone loss (cm)	Follow-up time (month)	Gustilo Classification
1	M/39	Traffic injury	6.0	20	IIIA
2	M/24	Crush injury	4.5	28	IIIA
3	F/24	Traffic injury	10.0	22	IIIA
4	M/41	Traffic injury	8.0	26	IIIA
5	F/34	Traffic injury	7.0	24	IIIA
6	F/27	Crush injury	9.5	21	IIIB
7	M/45	Traffic injury	7.5	25	IIIA
8	M/26	Traffic injury	9.0	27	IIIB
9	M/34	Traffic injury	7.5	34	IIIA
10	F/23	Traffic injury	5.5	23	IIIB

*M: male; F: female*

**Table 2:** Operative characteristics

Case	Wound area (cm^2^)	1^st^ operation fixation	Soft tisssue resonstruction	Three operation times (hours)	3^rd^ operation internal fixation
1	100	Composite external fixation frame	Posterior tibial artery perforator flap local transposition	4, 3, 2.5	Intramedullary nail + locking steel plate combined fixation
2	50		Sural nerve nutrient flap local transposition	2, 2.5, 2	Intramedullary nail internal fixation
3	180		Free anterolateral thigh flap	6.5, 4.5, 3.5	Intramedullary nail + locking steel plate combined fixation
4	120		Posterior tibial artery perforator flap local transposition	5, 3.5, 3	Intramedullary nail + locking steel plate combined fixation
5	70		Sural nerve nutrient flap local transposition	3, 2, 2.5	Intramedullary nail + locking steel plate combined fixation
6	150		Crossover posterior tibial artery perforator flap	6, 3.5, 3.5	Intramedullary nail + locking steel plate combined fixation
7	110		Sural nerve nutrient flap local transposition	4.5, 3, 3	Intramedullary nail + locking steel plate combined fixation
8	160		Crossover posterior tibial artery perforator flap	6, 4, 3.5	Intramedullary nail + locking steel plate combined fixation
9	130		Sural nerve nutrient flap local transposition	5.5, 3.5, 3	Intramedullary nail + locking steel plate combined fixation
10	80		Posterior tibial artery perforator flap local transposition	4.5, 2.5, 2.5	Intramedullary nail + locking steel plate combined fixation

*1* ^*st*^ *operation: Wound debridement + external fixation surgery*

*2*^*nd*^*operation: Cement filling + skin soft tissue defect skin flap repair surgery*.

*3*^*rd*^*operation: Induced membrane bone grafting + internal fixation*.

**Table 3:** Postoperative follow-up results

Case	Healing time (month)	Paley evaluation	AOFAS Ankle-Hindfoot Scale score	Complications
1	11	Excellent	83	None
2	11	Excellent	85	None
3	11	Good	74	Wound fat liquefaction with exudates
4	16	Excellent	81	None
5	11	Good	75	Bloated skin flap
6	11	Excellent	85	None
7	12	Good	77	Wound fat liquefaction with exudates
8	11	Excellent	84	None
9	13	Excellent	82	None
10	15	Good	79	None

## Discussion

The advancement of bone reconstruction using the Masquelet technique has progressed over more than twenty years, yet unfavorable results continue to be prevalent, particularly in tibial cases, potentially attributable to inadequate vascularity and reduced muscular envelopment in these regions [7]. Reports of Gustilo III open tibial fractures caused by high-energy injuries and subsequently managed utilizing the Masquelet technique were reviewed (Table 4). Sasaki et al. reported 1 Gustilo IIIA open tibial fracture with 5.5 cm of bone loss and the union time of 6 months [13]. Kang et al. reported 15 cases of Gustilo III open tibial fractures with an average union time ranging from 4 to 7 months [14]. Wang et al’s study revealed a mean union time of 6.1 months (range, 5 to 8 months) [9]. However, despite an even shorter average length of bone loss (5.3 and 5.1 cm), a much longer mean union time was indicated in the investigations of Zoller et al. and Lan et al. investigations (17.2 and 15.1 months, respectively) [15, 16]. In our current study, the union time of the fracture falls between the two groups, with an average union time of 12.2 months. Additionally, the average bone defect length in our study is 7.5 cm, which was greater than that in any of these reports, and no significant correlation was found between the length of bone loss and healing time (*r*=-0.164, *P* = 0.651). This finding is consistent with the findings of Masquelet, who reported that bone union time is not dependent on the length of bone loss [17, 18]. Notably, there was only one postoperative superficial infection in Kang’s study and two postoperative superficial infections in Wang’s study, whereas postoperative infections in Zoller and Lan’s study were associated with a high occurrence of postoperative osteomyelitis which suggested that a sterile surgical environment is a significant contributing factor to prolonged union time [15, 16]. However, in our study, the bone union time was relatively longer than that in Wang’s investigations. There are three possible explanations for this discrepancy: [1] Like the majority of studies that reported an average union time within one year using the Masquelet technique, the length of the bone defect was generally approximately 5 cm [16] [2]. This may be attributed to case-specific variations given the small sample size in this study [3]. The extensive duration of the study may also contribute to a potential bias in the surgical protocols.

**Table 4:** A literature review assessing Gustilo type III tibial fractures treated using the Masquelet technique

Publications	Gustilo III A, B, C	Age (years) mean (range)	Bone defect (cm) mean (range)	Healing time (months) mean (range)	Nonunion	Postoperative infection
Sasaki et al. 2017 [13]	1, 0, 0	39	5.5	6	0	0
Kang et al. 2020 [14]	0, 8, 7	46.5 (19–72)	5.8(4-11)	NA (4-7)	0	1
Wang et al. 2020 [9]	0, 0, 15	39.3 ( 21–43)	6.9 (5-11)	6.1 (5-8)	0	2
Zoller et al. 2017 [15]	0, 3, 0	34.3 (34–35)	5.3 (3-7)	17.2 (6-24)	2	2
Lan et al. 2022 [16]	0, 7, 8	43.7 (27–60)	5.1 (4-7)	15.1 (6-24)	2	8

The strategy for the treatment of Gustilo III open fractures includes thorough debridement, repair of vascular defects, bone coverage through plastic surgery procedures, bone fixation and reconstruction. This approach remains challenging for orthopedic and trauma surgeons. Microsurgical techniques combined with the Masquelet membrane induction technique provide desirable conditions for tibial salvage for open fractures in terms of both infection containment and bone regeneration. After complete debridement, negative-pressure treatment was applied in the first stage of the operation to prevent infection, and the anterolateral femoral flap as well as local flap, which aim for skin and soft tissue repair not only guaranteed good blood supply which assisted in infection control [19] but also enhanced membrane formation thereby providing an ideal microenvironment for subsequent bone grafting. An antibiotic-loaded cement spacer that allows continuous local antibiotic release further ensures better prevention of local infection. An analysis of multiple studies revealed that approximately 40% of patients required microsurgical techniques for soft tissue reconstruction [20]. Despite rare reports regarding the application of microsurgical techniques in open tibial fractures, Kang et al. and Wang et al. retrospectively studied patients treated with microsurgical techniques combined with the Masquelet technique for the reconstruction of fractures; these procedures achieved favorable outcomes, as indicated by short bone healing time and no deep infections [9, 14]. Here, despite the relative longer bone healing time, no deep infection or nonunion was observed and all patients achieved desirable bone function recovery. It is notewhorthy that the Masquelet technique is typically accomplished through two surgical interventions [21, 22], while in this study, debridement and external fixation were performed during the first surgery, with soft tissue reconstruction and bone cement implantation carried out during the second surgery. Then the bone grafting was employed in the third operation. This approach facilitates wound recovery and is more conducive to the formation of the inducing membrane in the later stages. Additionally, in the clinical reports of treatment for tibial defects [9, 14], free anterolateral thigh flaps were commenly used for soft tissue repair, while this study prioritized local flaps, considering free anterolateral thigh flaps only when wound coverage was difficult (only in one case with with a wound area of 180 cm^2^). Compared to free anterolateral thigh flaps, pedicled flap survival rates are high postoperatively, with lower risks of flap necrosis, thus favoring the wider adoption of this technique [23–25]. This study provides insight into the combined use of the Masquelet technique and microsurgery for the treatment of tibial fractures interms of different Gustilo types III tibial fractures and the utilization of local flaps for soft tissue repair, laying a foundation for further optimization and widespread application of subsequent methods.

At this stage, the cohort in our study was very small, and the data collection was retrospective without a control group, which limits the values of our findings, There is promise in the findings of this small-scale study that further prospective and comparative studies should be performed.

## Conclusion

We concluded that the use of the Masquelet technique in combination with microsurgical technique is a safe and effective strategy for the treatment of open tibial fractures with significant bone defects.

## Data Availability

The data used and/or analyzed during the current study are available from the corresponding author on reasonable request.
